# Effect of microplastics on the allelopathic effects of native and invasive plants on co-occurring invaders

**DOI:** 10.3389/fpls.2024.1425815

**Published:** 2024-10-28

**Authors:** Ling Yuan, Li Zhou, Junmin Li

**Affiliations:** ^1^ School of Advanced Study, Taizhou University, Taizhou, Zhejiang, China; ^2^ Zhejiang Provincial Key Laboratory of Plant Evolutionary Ecology and Conservation, Taizhou University, Taizhou, China

**Keywords:** polyethylene, germination, allelopathy, seedlings, metabolomics

## Abstract

**Introduction:**

Microplastic pollution has emerged as a significant global change factor, with the potential to alter the biological, physicochemical properties of soil and to subsequently affect plant growth. Despite growing recognition of the impacts of microplastic pollution, the mechanisms by which microplastics modify plant leaf chemistry and influence allelopathic interactions among co-existing plant species remain unclear.

**Methods:**

We used the native perennial forb *Achyranthes bidentata* and the invasive annual forb *Amaranthus spinosus* as focal species. We grew the two species with and without competition with each other. This setup was further combined with a treatment involving the addition of polyethylene (PE). We then testd the effects of aqueous extract on seed germination and seedling growth for five invasive and five native species. Subsequently, metabolomic analysis was conducted on the aqueous extracts, in which significant allelopathic effects were observed on test species.

**Results and discussion:**

The presence of PE microplastics enhanced the biomass of both *Achyranthes* and *Amaranthus* under competitive and non-competitive growth conditions. Furthermore, PE microplastics were found to induce a negative allelopathic effect for the native plant *Achyranthes* on co-occurring plants, which appeared to be mediated through changes in leaf chemistry. Bisdemethoxycurcumin, ethylparaben, salicin 6’-sulfate and 5-hydroxy-3’,4’,7-trimethoxyflavone glucoside were proven important compounds for allelopathic enhancement. Overall, these results suggest that microplastic pollution has the capability to influence the co-existence of invasive and native plants by altering their allelopathic potential. This insight into the interactions between microplastics and plant allelopathy provides a novel perspective on how microplastic pollution could modify plant species interactions and ecosystem dynamics. Future studies could aim to answer how microplastics might affect plant root exudates and whether this process would mediate biological invasion.

## 1 Introduction

Invasive plant species are a global concern due to their ability to out-compete native vegetation, disrupting the balance of ecosystems and then leading to a decline in native species populations (Vila and Hulme, 2017; Li et al., 2024). While invasive plants alter the attributes of ecosystems, human activities such as deliberate planting, habitat degradation, climate change and environmental pollution often promote their colonization and further facilitate the invasion success (Prabakaran et al., 2019; Li et al., 2023; Iqbal et al., 2024). By 2030, environmental pollution (including plastic pollution) has been predicted to increase continuously and sharply, according to an assessment released by the UN Environment Programme (UNEP). Therefore, it is imperative to understand how both invasive and native plants response to environmental pollution stress in invaded ecosystems, in order to restore biodiversity and develop effective strategies to manage and mitigate the impacts of invasive species on native flora.

In recent years, the environmental stress induced by microplastic pollution has emerged as a major factor in the balance of ecological health and sustainability. However, we still lack a comprehensive understanding of global implications regarding microplastics pollutions (Rillig, 2012; de Souza MaChado et al., 2019). Microplastics, defined as plastic particles with dimensions ranging from a nanometer to a micrometer scale (<5 mm), have become pervasive contaminants in both terrestrial and aquatic ecosystems. The ubiquitous nature of microplastic pollution is due to their widespread sources, including the breakdown of commercial and industrial plastic wastes, and microbeads in personal care products and fibers from synthetic textiles (Rillig, 2012). As an emerging type of environmental pollutant, microplastics can change the physical properties of soil and have a serious impact on soil function (Leed and Smithson, 2019). For example, microplastics can increase soil porosity and adsorb pollutants present in the soil solution, thus exert major effects on soil ecosystems (Kim et al., 2021).

Microplastics pose significant risks to terrestrial systems owing to their high abundance and strong persistence. The potential effects of microplastics on plant growth could be divided into direct mechanisms, such as blocking stomata, causing mechanical damage to roots, downregulating gene expression or releasing additives, and indirect mechanism, such as changing soil properties, affecting soil microbes or soil fauna, and affecting bioavailability of other contaminants (Li et al., 2022). Recent studies have begun to identify the subtle yet significant effects of microplastics on plant physiology, growth and health, with results indicating a complex range of interactions (de Souza MaChado et al., 2019; Lozano et al., 2021a, b). However, few studies have focused on the comparative responses of invasive and native plants to microplastics, or the resulting ecological impacts.

Allelopathy refers to the ability of plants to release biochemicals that influence the growth and development of surrounding organisms, which is a widely recognized ecological phenomenon affecting species interactions, community structure, and ecosystem function (Zhang et al., 2021). Allelopathic compounds are released into the environment through volatilization, diffusion, leaching from above-ground plant regions, root exudation, and litter decomposition (Chomel et al., 2016). The composition and concentration of plant allelochemicals can be altered by abiotic factors, such as light and climate warming (Sun et al., 2022), as well as by biotic factors such as competition (Yuan et al., 2022) and herbivory predation (Dayan, 2006; Kong et al., 2006; Song et al., 2008; Kato-Noguchi, 2011). Although microplastic pollution has emerged as a significant environmental modifier, it remains unclear whether microplastics can alter the allelopathic effects of invasive and native plants on co-occurring plants.

In this study, a greenhouse experiment was performed to determine the different responses of two sympatric plant species, native *Achyranthes bidentata* (hereafter referred to as *Achyranthes*) and invasive *Amaranthus spinosus* (hereafter referred to as *Amaranthus*), to the addition of polyethylene (PE) microplastics under both competitive and non-competitive growth conditions. A controlled multi-species germination experiment was performed to assess the allelopathic effects of aqueous leachates from both invasive and native plants, with the aqueous leachates subsequently subjected to comprehensive metabolomic profiling. The aim of this research was to identify the effects of microplastics on allelopathic interactions between invasive and native plants, focusing on: (1) how microplastics affect plant invasion by changing the growth and competitive abilities of invasive or native species; and (2) how their allelopathic effects influence the germination of invasive and native species. We hypothesized that PE powder could change leaf chemistry of both alien *Amaranthus* and native *Achyranthes* and thereby affect their allelopathy potential. By integrating the impacts of microplastic pollution into the broader framework of invasion ecology, this study advances our understanding of how emerging pollutants can influence biological invasions.

## 2 Materials and methods

### 2.1 Species selection

In order to avoid a confounding of status with phylogeny, the invasive annual forb *Amaranthus* and the native perennial forb *Achyranthes* belong to the Amaranthus and Achyranthe genera of the Amaranthaceae family, respectively. These species commonly co-occur in various habitats throughout Zhejiang province, particularly in disturbed areas (Zhang and Ding, 1993). Both *Amaranthus* and *Achyranthes* were reported to be allelopathic (Carvalho et al., 2019; Yuan et al., 2021)*. Amaranthus* has gained attention as one of the most harmful invasive species in China due to its capability to significantly impair the function of ecosystems, as recognized by the Ministry of Environmental Protection of the People’s Republic of China in 2010. In East China, *Amaranthus* species account for 5.35% of all invasive species, with this proportion exceeding any other invasive species in the region (Yan et al., 2021).

In total, 10 herbaceous plants were selected as test species for the allelopathic study, all of which naturally coexist in a diverse range of habitats across eastern China, as documented by Zhang and Ding (1993). The test group comprised an equal representation of both invasive (*n*=5) and native (*n*=5) species (**Supplementary Table S1**, Supporting Information), allowing for an assessment of the influence of microplastics on allelopathic interactions. All the seeds were purchased from a commercial seed supplier (Thousand Green Seed Company, Jiangsu, Suqian, China).

### 2.2 Experiment set up

PE, a common type of microplastic in all environments and widely present in sewage sludge used in agriculture (Souza MaChado et al., 2018b), was selected for the study. To determine whether microplastics affect the biomass of plants under competitive and non-competitive conditions, three-factor two-level random factorial experiments were conducted. The fixed terms in these experiments included species origin (invasive or native), competition (competitive free/interspecific competition), microplastics (exposed or non-exposed) and their interaction.

On June 15th 2020, we germinated the seeds on plastic containers filled with a 1:1 mixure of sand and vermiculite in a growth chamber (day-time temperature: 18-21°C; night-time temperature: 16-20°C; day length: 14 hours; relative humidity: 60%). On June 30^th^ 2020, the germinated seedlings were transplanted into 2 L plastic pots. Each pot was filled with 1.45 kg soil and 9.7 g PE, resulting a 1% PE in mass ratio (which as reported in previous research Souza MaChado, 2018a). The soil was collected from 4 locations in the mountainous area of Taizhou City (Zhejiang, China). The mixed soil was sandy loam and had a pH of 4.95, an organic matter content of 15.65 g kg^-1^, a total nitrogen content of 0.14 g kg^-1^, an available phosphorus content of 36.12 mg kg^-1^ (extracted using sodium bicarbonate), and an available potassium content of 162.32 mg kg^-1^ (extracted using ammonium acetate). PE powder with 1000 mesh and 13 µm particle diameter was purchased from Zhongxin Plastic Co. Ltd., Shenzhen, China.

For each plant species, four separate treatments were established, consisting of (1) a non-competitive system with a single plant per pot, (2) an interspecific competitive system with two plants (one of each species) per pot, (3) non-competitive system as described in (1) with PE microplastics exposure and (4) a competitive system as described in (2) with PE microplastics exposure. For each treatment, 12 pots were used (*n*=48) as replicates. The pots were then placed in a greenhouse under controlled conditions with daytime temperatures of 22-25°C, overnight temperatures of 18-21°C, a 14 hr photoperiod and a relative humidity of 60%.

On September 27^th^, 2020, we harvested the plants for biomass measurement and the preparation of aqueous extracts. Plants were washed to remove growth medium and then leaves were collected from each individual, which were subsequently oven-dried at 70°C. The above-ground biomass of each plant was calculated. To quantify the competitive effects and responses of *Achyranthes* and *Amaranthus*, relative interaction index (RII) values were computed using the above-ground biomass, following the method described by Armas et al. (2004).

### 2.3 Preparation of aqueous extracts and evaluation of allelopathic effects

For each treatment, four individual plants were mixed to prepare one sample of aqueous extract samples, resulting three aqueous extracts per treatment. For each sample, 30 g of dry leaves were placed in beakers containing 1000 mL of distilled water, then left to soak for 24 h at room temperature, allowing the extraction of substances which may affect the germination of test seeds (Butcko and Jensen, 2002; Pisula and Meiners, 2010). Each extract was initially filtered (Whatman No.1 filter paper) and then through a 0.8 μm filter membrane (25 mm in diameter) to remove fungal spores. The aqueous extract was then stored in a separate sterilized centrifuge tube in the dark at -40°C until use.

To evaluate the allelopathic potential of the prepared aqueous *Achyranthes* and *Amaranthus* extracts on seed germination, a multiple-species germination experiment was conducted. A total of 270 Petri dishes (6 cm diameter) were prepared, corresponding to 10 test species, 3 replicates each and 9 different treatments: 1) control; 2) *Achyranthes* (no competition) without microplastics; 3) *Achyranthes* (no competition) with microplastics; 4) *Achyranthes* (interspecific competition) without microplastics; 5) *Achyranthes* (interspecific competition) with microplastics; 6) *Amaranthus* (no competition) without microplastics; 7) *Amaranthus* (no competition) with microplastics; 8) *Amaranthus* (interspecific competition) without microplastics; 9) *Amaranthus* (interspecific competition) with microplastics. Petri dishes were filled with a mixture of plant extract and agar gel at a 1:2 volume ratio. The agar gel was prepared by combining 12 g of high-strength agar, 30 g of sucrose and 3.225 g of Murashige and Skoog medium nutrients in 1 liter of distilled water. The pH of the agar gel was adjusted to 6.0 using NaOH and HCl. The agar medium underwent sterilization for 15 minutes at 120°C and a pressure of 100 kPa, then was transferred to a 30°C water bath to maintain its liquid state. The plant extracts were thawed in a heating cabinet at 30°C for 30 minutes and subsequently filtered through 0.8 μm filter membranes to ensure sterility. As a baseline control, 30 Petri dishes were filled with agar gel without any plant extracts. As the osmolality of the germination medium can significantly influence seed germination (Inderjit and Nilsen, 2003; Oduor et al., 2020), the osmolality of all plant extracts was measured using an osmometer (Wescor 5600, Shanghai Pengqi Scientific Instrument Co. Ltd., China). To standardize osmolality for all treatments, the osmolality of the control treatments agar solution was adjusted to match the mean value of the plant extract agar solutions, which was achieved by incorporating an 8000-polyethyleneglycol (PEG; Sigma-Aldrich, Germany) solution into the agar, resulting in a final concentration of 0.023 g PEG/mL.

On the 8^th^ November 2020, 10 seeds of each of the 10 test species were introduced into the 270 prepared Petri dishes. Prior to planting, the seeds were sterilized by immersion in a 5% sodium hypochlorite solution for 5 minutes, followed by a thorough rinsing with distilled water to remove any residues. Once the seeds were placed in the petri dishes, they were sealed with parafilm to prevent losing water and then placed within a phytochamber to ensure uniform conditions. The germination process of each petri dish was monitored and recorded daily. The experiment was concluded on 22^nd^ November 2020, approximately 14 days after the emergence of the first seedling and five days after the last seedling had germinated.

### 2.4 Metabolomic analysis of plant extracts

As the germination experiment suggested that PE exposure significantly altered the allelopathic effects of native *Achyranthes*, six samples were submitted for metabolomic analysis to determine whether PE microplastics altered the phytochemical composition of plants. These six samples consisted of three replicate samples of aqueous extracts of (1) *Achyranthes* cultivated without competition and without PE microplastics, (2) *Achyranthes* cultivated without competition and with PE microplastics. The analysis was performed by Wuhan Metware Biotechnology Co. Ltd. (Wuhan, China). The samples were processed using an ultra-performance liquid chromatography-tandem mass spectrometry (UPLC-ESI-MS/MS) system (Nexera X2, Shimadzu; Applied Biosystems 4500 Q TRAP). The protocols followed the general pipeline in Wuhan Metenware Biotechnology Co. Ltd. (China), with certain adjustments when necessary (Chen et al., 2013).

### 2.5 Statistical analysis

All statistical analysis was performed using R software v. 4.0.3 (R Core Team 2020), unless stated otherwise.

A linear model was used to determine whether species origin, competition and microplastic exposure during cultivation affected the biomass of *Amaranthus* and *Achyranthes* plants. The explanatory terms included the origin of test species (invasive/native), microplastic (the presence/absence of microplastics) and competition (competitive free/interspecific competition), as well as their interactions.

To analyze the germination rate of seeds and the root length of the initial seedling a binomial generalized linear mixed model (GLMM) and a linear mixed model (LMM) were used, respectively. The models incorporated the ‘origin’ (invasive or native) and ‘extract type’ (control; Achy: *Achyranthes*; Amar: *Amaranthus*; Achyfree: *Achyranthes* without competition; Achyinter: *Achyranthes* with interspecific competition; Amarfree: *Amaranthus* without competition; Amarinter: *Amaranthus* with interspecific competition; WithAmarfree: *Amaranthus* with microplastics but without competition; WithAmarinter: *Amaranthus* with microplastics and interspecific competition; WithoutAmarfree: *Amaranthus* without microplastics or competition; WithoutAmarinter: *Amaranthus* without microplastics but with interspecific competition as fixed effects, along with their potential interactions. Variation among test species was treated as a random effect. To further scrutinize the differences among the nine aqueous extract treatments, eight orthogonal contrasts were formulated for ‘extract type’. The initial contrast assessed the overall impact of plant extracts by comparing the polyethylene glycol (PEG) control to average of all plant extracts. The second contrast evaluated differences between the averages of *Achyranthes* and *Amaranthus* extracts. The third contrast evaluated differences between *Achyranthes* with and/or without microplastic. The fourth contrast evaluated differences between *Amaranthus* with and/or without microplastic. The fifth contrasts evaluated differences between with microplastic addition, *Achyranthes* without competition and with interspecifc competition. The sixth contrasts evaluated differences between without microplastic addition, *Achyranthes* without competition and with interspecifc competition. The seventh contrasts evaluated differences between with microplastic addition, *Amaranthus* without competition and with interspecifc competition. The eighth contrasts evaluated differences between without microplastic addition, *Amaranthus* without competition and with interspecifc competition. To enhance the normality of residuals in the root length analysis, log transformation was applied. Heterogeneity of variance was addressed by allowing variance to differ between the two test species and among the extract types, using the varComb and varIdent functions within the lme function of the nlme package (Pinheiro et al., 2020; Zuur et al., 2009). The significance of fixed effects and contrasts were determined using log-likelihood ratio tests, involving a comparison of model outputs with and without the term of interest, as described by Zuur et al. (2009).

Principal component analysis (PCA), an unsupervised statistical technique, was executed using the prcomp function of R base. Prior to PCA, the dataset was preprocessed with unit variance scaling to normalize variables. In the context of a two-group comparison, differential metabolites were identified based on their variable importance in projection (VIP) scores, with a threshold of VIP > 1 and absolute Log_2_ fold change (|Log_2_FC|) values greater than or equal to 1. The VIP values were derived from the results of orthogonal partial least squares discriminant analysis (OPLS-DA), which also provided score plots and permutation plots, which were generated using the MetaboAnalystR package in R. Before conducting OPLS-DA, the data was subjected to log transformation and mean centering to ensure appropriate analysis. To prevent overfitting, a permutation test was conducted with 200 iterations. The identified metabolites were annotated against the KEGG compound database. Following annotation, these metabolites were mapped onto their corresponding metabolic pathways within the KEGG pathway database, for a comprehensive understanding of their biological significance and network of involvement in different metabolic processes.

## 3 Results

### 3.1 Impact of microplastics on the competitive ability of plants

**Figure 1:**
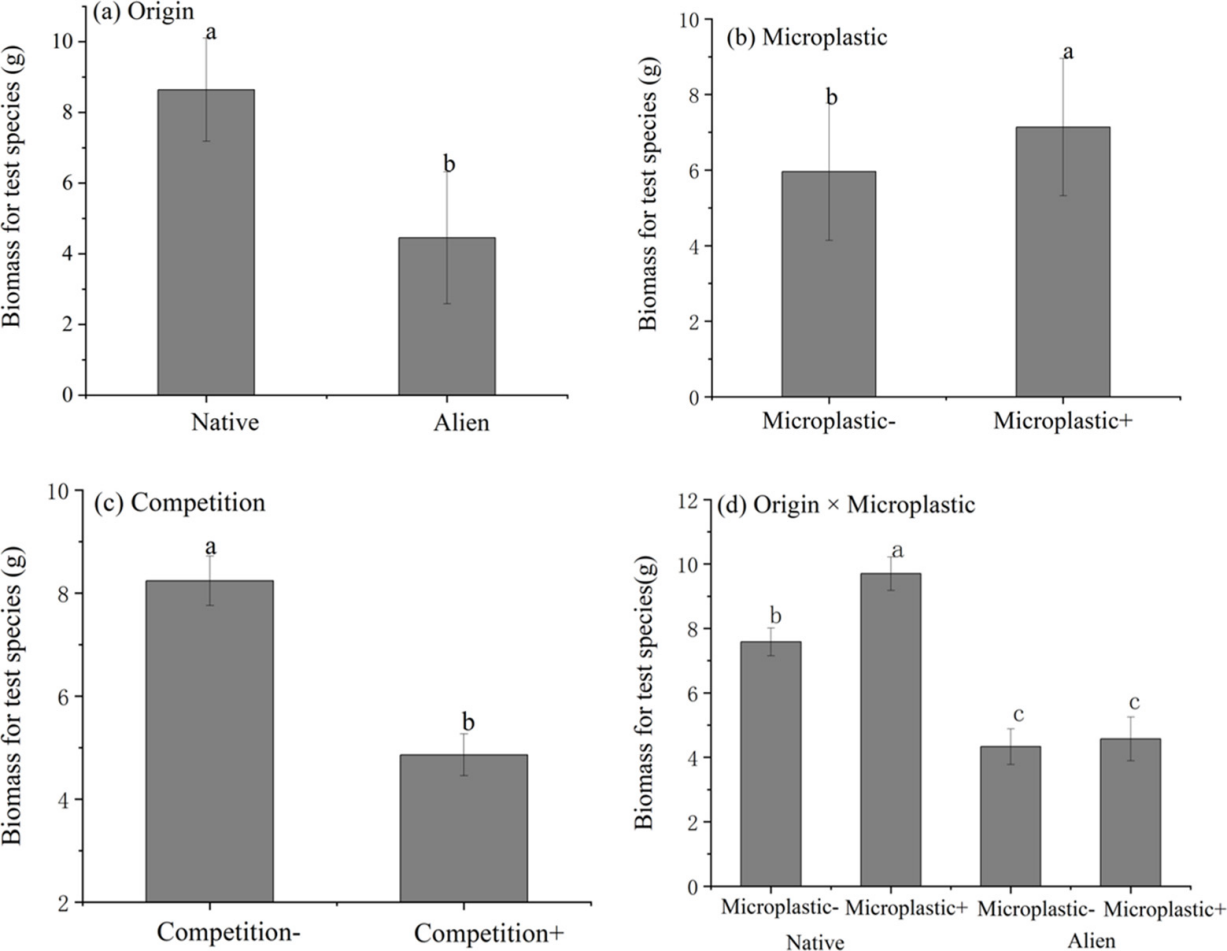
Boxplots showing the effects of competition and microplastics exposure on the above-ground biomass for *Achyranthes bidentata* and *Amaranthus spinosus*. **(A)** Effects of origin of test species on biomass; **(B)** Effects of microplastics on biomass; **(C)** Effects of competition on biomass; **(D)** The interaction effects of origin and microplastics on biomass. Letters above the bars indicate the results of Sidak post-hoc comparisons; bars that do not share a letter are significantly different from each other (P < 0.05).

For *Achyranthes* and *Amaranthu*s, above-ground biomass was found to vary according to competition conditions and the test species origin (**Figure 1**; **Table 1**). Exposure to microplastics had a significant effect on individual plant biomass, although this effect was depending on the test species being invasive or native. For the native species (*Achyranthes*), microplastics significantly increased above-ground biomass by 27.9%, irrespective of whether the plant was cultivated with or without competition. For the invasive test species (*Amaranthu*s), exposure to microplastics had no obvious effect on above-ground biomass. Nevertheless, exposure to microplastics had no significant effect on the competitive ability (RII) of either *Achyranthes* or *Amaranthu*s (**Supplementary Table S3**, Supporting Information).

**Table 1:** Results of a linear model testing the effect of origin (origin of test species), competition (with/without) and microplastics (with/without) on individual plant specimen biomass at the end of the competition experiment.

Factors	df	F	*P*
origin	1	60.454	< 0.001
microplastic	1	11.965	0.007
competition	1	51.953	< 0.001
origin*microplastic	1	5.366	0.021
origin*competition	1	0.404	0.525
microplastic*competition	1	2.337	0.126
microplastic * microplastic * competition	1	0.487	0.485

### 3.2 Impact of microplastics on plant allelopathy

**Figure 2:**
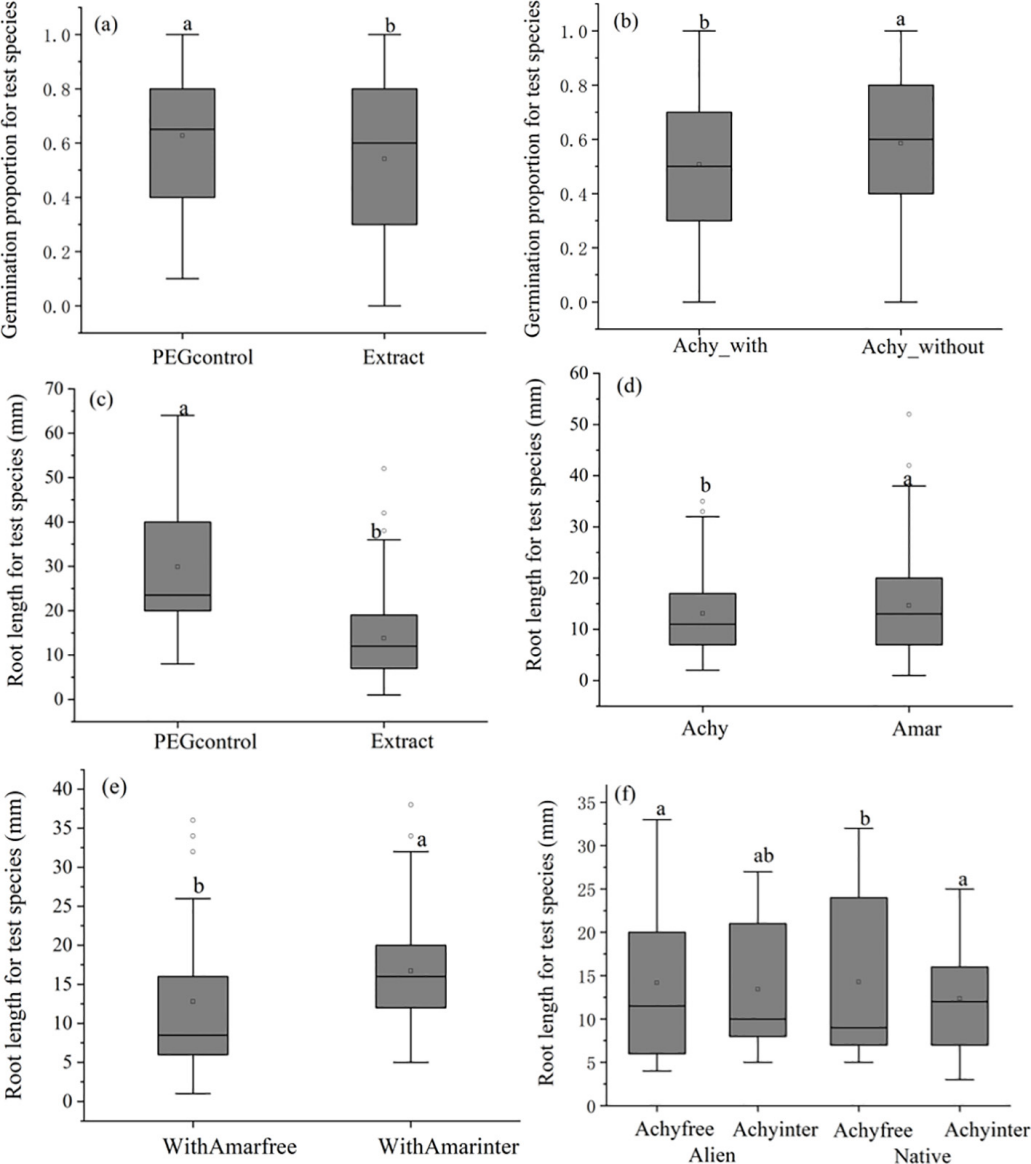
Boxplots showing the effects of *Achyranthes bidentata* and *Amaranthus spinosus* aqueous extracts following different treatments on the germination performance and root length of 14 day-old seedlings. **(A)** The effects of extract on germination rate; **(B)** The effects of Achy with microplastic on germination rates; **(C)** The effects of extract on root length; **(D)** The effects of the two allelopathy plant on root length; **(E)** The effect of inter-specific competition for Amar with microplastic on root length; **(F)** Interaction effects of interspecific competition and allelopathy plant origin on root length. Test species included Achy: *Achyranthes bidentata*, Amar: *Amaranthus spinosus*, Achyfree: *Achyranthes bidentata* without competition, Achyinter: *Achyranthes bidentata* with interspecific competition, WithAmarfree: *Amaranthus spinosus* with microplastics but without competition, WithAmarinter: *Amaranthus spinosus* with microplastics and interspecific competition. Letters above the bars indicate the results of Sidak post-hoc comparisons; bars that do not share a letter are significantly different from each other (P < 0.05)

Across all experimental treatments, there were no significant differences observed in the overall rate of germination of invasive and native test species seeds. However, the presence of aqueous plant extracts significantly decreased the proportion of germinated seeds by 13.7% (**Figure 2A**). Germination proportion in extracts from *Achyranthes* plants cultivated in the absence of microplastics was 15.2% higher than extracts from plants grown in the presence of microplastics (χ2 = 9.7, p= 0.0018, **Figure 2B**; **Table 2**). This effect was not found in *Amaranthus* plant extracts (χ2 = 3.757, p=0.153, **Table 2**).

**Table 2:** GLM results for germination rates and LMM for root length.

	df	Germination rate		root length	
		χ^2^	P	χ^2^	P
Fixed effects					
Origin	1	0.0277	0.868	0.0019	0.966
Extract type	8	26.811	<0.001	157.62	<0.001
Control_extract	1	9.468	0.002	149.25	<0.001
Achy-Amar	1	0.0063	0.937	4.0558	0.044
Achy with_without	1	9.700	0.0018	0.001	0.992
Amar with_without	1	3.757	0.153	0.004	0.947
With_Achyfree-inter	1	0.408	0.522	3.147	0.076
Without_Achyfree-inter	1	0.226	0.635	0.106	0.744
With_Amarfree-inter	1	0.3096	0.578	11.757	<0.001
Without_Amarfree-inter	1	3.551	0.059	0.422	0.516
Origin×Extarct type	8	4.7124	0.787	6.349	0.608
O×[Control_extract]	1	0.247	0.619	2.099	0.147
O×[Achy-Amar]	1	0.958	0.328	0.09	0.746
O×[Achy with_without]	1	0.743	0.389	0.135	0.713
O×[Amar with_without]	1	0.789	0.374	0.001	0.973
O×[With_Achyfree-inter]	1	0.187	0.665	4.343	0.037
O×[Without_Achyfree-inter]	1	0.042	0.838	1.188	0.258
O×[With_Amarfree-inter]	1	1.776	0.183	1.877	0.171
O×[Without_Amarfree-inter]	1	0.018	0.894	0.341	0.559
Radom effects		SD		SD	
Test species	9	1.046		6.974	

Achy, *Achyranthes bidentata*; Amar, *Amaranthus spinosus*; Achyfree, *Achyranthes bidentata* without competition; Achyinter, *Achyranthes bidentata* with interspecific competition; Amarfree, Amaranthus spinosus without competition; Amarinter, *Amaranthus spinosus* with interspecific competition; WithAmarfree. *Amaranthus spinosus* with microplastics but without competition; WithAmarinter, *Amaranthus spinosus* with microplastics and interspecific competition; WithoutAmarfree, *Amaranthus spinosus* without microplastics or competition; WithoutAmarinter, *Amaranthus spinosus* without microplastics but with interspecific competition.

For all test species, root length was significantly reduced by 52.9% when exposed to plant extracts, comparing to the PEG control (**Figure 2C**; **Table 2**). Under conditions of microplastics exposure, the extracts of *Amaranthus* plants grown without competition exerted a 23.6% greater inhibition effect on root length, than extracts of *Amaranthus* plants grown with interspecific competition (**Figure 2E**). In contrast, extracts from *Achyranthes* cultivated with microplastics had varying impacts on root length depending on whether the test species was invasive or native (**Figure 2D**). For alien test species, extracts from *Achyranthes* cultivated with interspecific competition decreased root length by 5.6% compared to extracts from *Achyranthes* cultivated under non-competitive conditions. Conversely, for native test species, extracts of *Achyranthes* cultivated with interspecific competition resulted in a 15.4% increase for root length (**Figure 2F**).

### 3.3 Impact of microplastics on the metabolomic profile of plants

**Figure 3:**
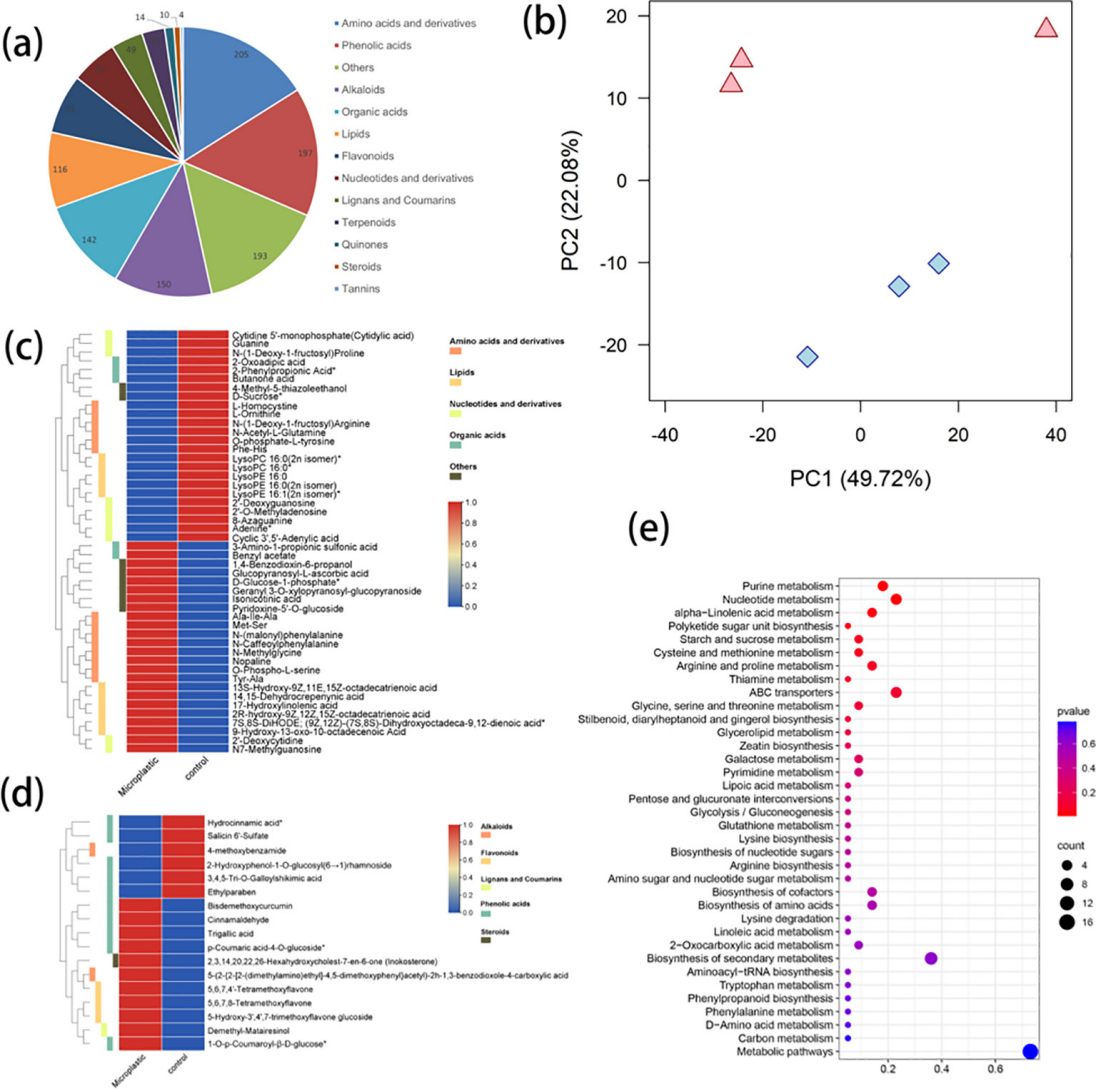
Quality control of metabolomics data and changes in differentially accumulated metabolites (DAMs) between *Achyranthes bidentata* with/without microplastics exposure: **(A)** All metabolites identified in all samples; **(B)** Results of unsupervised principal component analysis of metabolomics data, showing scores along the first two PC axes. Samples of *Achyranthes bidentata* exposed to microplastics are shown in pink, while samples of *Achyranthes bidentata* without microplastics exposure are shown in blue; **(C)** heatmap of primary metabolites; **(D)** heatmap of secondary metabolites; **(E)** KEGG pathways enriched for DAMs.

Metabolomic profiling was performed on aqueous extracts from *Achyranthes* cultivated in both the presence or absence of microplastics, detecting 1277 distinct metabolite compounds. All of the 1277 metabolites were identified in all samples, which were divided into 13 categories, including 205 amino acids/derivatives (16.05%), 197 phenolic acids (15.43%), 193 others (15.11%), 150 alkaloids (11.75%), 142 organic acids (11.12%), 116 lipids (9.08%), 91 flavonoids (7.13%), 71 nucleotides/derivatives (5.56%), 49 lignans/coumarins (3.84%), 35 terpenoids (2.74%), 14 quinones (1.10%), 10 steroids (0.78%) and 4 tannins (0.31%) (**Figure 3A**). The results of PCA showed that samples from *Achyranthes* cultivated in the presence and absence of microplastic exposure were separated clearly into PC1 (49.72%) and PC2 (22.08%) (**Figure 3B**), indicating an obvious difference in the metabolic profile of these samples. In addition, a total of 65 metabolites were found to be differentially accumulated depending on the treatment conditions, including 48 primary metabolites (**Figure 3C**) and 17 secondary metabolites (**Figure 3D**). Specially, the accumulation of 24 primary metabolites was increased, including 8 amino acids/derivatives, 6 lipids, 2 nucleotides/derivatives, 2 organic acids and 6 other metabolites, while the accumulation of 24 primary metabolites was reduced, including 8 nucleotides/derivatives, 6 amino acids/derivatives, 5 lipids, 3 organic acids and 2 other metabolites (**Figure 3C**). Among the differentially accumulated secondary metabolites, the accumulation was increased for 11 (5 phenolic acids, 3 flavonoids, 1 alkaloid and 1 steroid) and accumulation was reduced for 6 (5 phenolic acids and 1 alkaloid) (**Figure 3D**). Furthermore, the top 20 differential accumulation metabolites (DAMS) exhibiting differences in abundance in *Achyranthes* cultivated with or without PE microplastics exposure, were identified (**Supplementary Figure S1**; **Supplementary Table S1**). Among these were 3 phenolic acids (bisdemethoxycurcumin, Log_2_ FC=1.49, ethylparaben, Log_2_ FC=-1.99 and salicin 6’-sulfate, Log_2_ FC=-3.41) and 1 flavonoid (5-Hydroxy-3’,4’,7- trimethoxyflavone glucoside, Log_2_ FC=1.96), which may play important roles in the enhancement of allelopathy in *Achyranthes* (**Supplementary Table S1**, Supporting Information). Furthermore, the results of KEGG enrichment analysis showed that the DAMs were enriched in 37 pathways, among which the most enriched pathways included metabolic pathways (16 metabolites), secondary metabolite biosynthesis (8 metabolites), nucleotide metabolism (5 metabolites), ABC transporters (5 metabolites) and purine metabolism (4 metabolites) (**Figures 3C, D**). Most DAMs were mapped onto “Purine metabolism”, “Nucleotide metabolism”, “Alpha Linolenic acid metabolism”, “Starch and sucrose metabolism”, “Argine and proline metabolism” and “Glycine, serine and threonine metabolism” pathways, among others (**Figure 3E**).

## 4 Discussion

The results of this study show that PE microplastics could significantly increase the above-ground biomass of both native *Achyranthes* and invasive *Amaranthus* when cultivated without competition, while only native *Achyranthes* exhibited an increase in above-ground biomass when cultivated under competitive conditions. However, PE microplastics had no significant effect on the competitive performance (RII, **Supplementary Table S3**) of either *Achyranthes* or *Amaranthus.* Furthermore, results indicated that PE microplastics had the capacity to amplify the allelopathic effects exerted by native *Achyranthes* on various invasive and native species, while this phenomenon was not observed with invasive *Amaranthus*. Metabolomic analysis of aqueous plant extracts showed that *Achyranthes* plants cultivated in the presence and absence of PE microplastics exhibited distinct chemical profiles, indicating the modifying impact of PE on their metabolic profiles. The results supported our hypothesis that PE might change plant allelopathy potential by changing leaf chemistry but only for native *Achyranthes*. Although this study only investigated the responses of two species, these results clearly suggest that PE microplastic pollution had no significant effect on the invasiveness of the invasive species, while supporting the survival of the native species in the presence of invasive species by enhancing the native plants allelopathic response.

Various investigations have established that the presence of microplastics in soil exerts a considerable influence on plant biomass (Li et al., 2024; Khan et al., 2024; Iqbal et al., 2024). However, the implications of microplastic exposure vary remarkably depending on the plant species and organs exposed, highlighting the complexity of the effect. For example, when plants such as onions and lettuce were exposed to microplastics, a significant reduction was observed in root biomass and above-ground biomass, while this exposure stimulated the growth of below-ground biomass in ryegrass (de Souza MaChado et al., 2019; Lozano and Rillig, 2020; Gao et al., 2021). In the present study, the consistent increase in *Achyranthes* biomass in response to PE microplastics implies that PE microplastics had a direct and positive influence on the growth dynamics of *Achyranthes*. It was observed that *Amaranthus* also exhibited a minor (not significant) increase in biomass under non-competitive conditions when exposed to PE microplastics, suggesting that PE may have a general growth-promoting effect on plants. However, this effect was not observed in *Amaranthus* under competitive conditions, which may be attributed to the interactions of various factors such as plant physiological characteristics, conditions within the rhizospheric environment, and the type and concentration of PE microplastics (Li et al., 2023). Microplastics may modulate plant growth through various mechanisms, including improving water retention, positively affecting water dynamics and subsequently enhancing photosynthetic efficiency (Souza MaChado, 2018a; Bender et al., 2016). The increased water-holding capacity provided by microplastics can increase soil moisture levels, enhancing nutrient availability by altering chemical speciation processes within the soil or influencing the activity of soil microbes (Souza MaChado, 2018a). Previous studies have indicated that microplastics can affect the abundance of arbuscular mycorrhizal fungi (AMF), which are crucial components of the soil system for nutrient exchange and plant growth (Bender et al., 2016). Mycorrhizal symbiosis can improve nutrient uptake, potentially contributing to the biomass increase observed in PE-exposed plants. Also, the microplastic precipitates might affect the growth of plants through blocking light or degrade in the leaves and release toxic substances (Li et al., 2022). For example, aggregation of microplastic particles in the leaf vessels might block cell junctions or cell wall pores (Sun et al., 2021). However, a short-term greenhouse experiment could rule out the possibility that atmospheric transport of PE would influence our results.

The intensification of allelopathic effects by *Achyranthes* when cultivated in a PE contaminated environment was significant, with the observed increase in allelopathic potential indicating that this is a strategic stress response by native plants, with secondary metabolites acting as nutrient chelators, making nutrients accessible to plants (Thelen et al., 2005). Additionally, PE might alter the soil microbial community, which has been shown to induce chemical changes in plant leaves (Moore et al., 2003; Giron et al., 2013; Kos et al., 2015). For example, it has previously been reported that soil microbial communities can alter leaf chemistry and influence the allelopathic potential of coexisting plant species (Meiners et al., 2017). The observation that PE microplastics enhance the allelopathic effects of native *Achyranthes* suggests that microplastics may interact with allelochemicals produced by these plants or alter the environmental conditions, making these chemicals more effective against invasive species. The fact that this phenomenon was not observed in the invasive species *Amaranthus* implies that certain responses to the presence of microplastics may be species-specific. The impact of soil microplastics on plants depends on the combined effect of various factors, including environmental conditions, the accumulation or transmission of microplastics within plant tissues, and species differences (Li et al., 2023).

Metabolomics analysis revealed the modification of chemical profiles in PE-exposed *Achyranthes*, providing insights into the molecular mechanisms involved. Results showed that bisdemethoxycurcumin, ethylparaben, salicin 6’-sulfate and 5-hydroxy-3’,4’,7-trimethoxyflavone glucoside may be important compounds for determining the degree of allelopathic enhancement. Salicin has been confirmed as an allelopathic compound in *Populus* L (Li, 2019). KEGG enrichment analysis indicated some DAMs were mapped onto argine, proline glycine, serine and threonine metabolism, which are reported to play an important role for plant enhancing resistance to adverse conditions (i.e. drought, salinity stress, low temperature etc.). These results indicated that *Achyranthes* might suffer from stress when exposed to microplastic. According to a recent research, microplastic could induce drought for plant due to increased soil cracking effects (Jia et al., 2023). PE might induce some drought stress for *Achyranthes* in our study. The enriched pathway for starch and sucrose metabolism are indispensable parts of plant growth, development, and biomass accumulation. By regulating these metabolic pathways, the growth performance and increase in biomass of plants can be affected (Li et al., 2017; Jeandet et al., 2022). PE might enhance starch and sucrose metabolism thereby enhanced the biomass. In future research, a correlation analysis between germination results and metabolites might give more insights for similar research.

Research on allelopathy is challenging, and a wide variety of experimental approaches have been used to test for allelopathic interactions. Like some allelopathy studies (Li et al., 2021; Zhang et al., 2021; Đorđević et al., 2022), we used leachates from plant leaves that had been cultivated in a controlled environment with benign greenhouse conditions. As the 10 test species were selected on the basis that they occur in a similar distribution range of *Achyranthes* and *Amaranthus* in China (Zhang and Ding, 1993), there are possibilities that *Achyranthes* and *Amaranthus* might affect their germination in nature (i.e. in rainy season). Several studies reported allelopathic effects of root exudates and some studies found potential allelochemicals in aboveground tissues (Abhilasha et al., 2008; Kato-Noguchi and Kato, 2022). However as we made the extracts of leaves of the allelopathic plants, we exclude the effects of compounds produced by the roots. On the other hand, as our plant were cultivated in plastic pots, plants in the control treatments without experimental microplastic addition might due to abrasion have been exposed to microplastic particles of the pots. Though the soil we used was collected from a mountain area in taizhou, a lately research reported that remote mountainous area inevitably becomes temporal sink for microplastics driven by atmospheric transport (Wei et al., 2024). There is possibilities the soil we used could have minor microplastic by air microplastic precipitation. Further research on this topic thus should perform experiments use pots not made of plastic (e.g. clay pots) and using soil without microplastic pollution to deepen our understanding of the effects of microplastics on plants. This caveat should not be ignored when interpreting the results of our study and those of other studies on allelopathy. Future studies could focus on how microplastics might affect plant root exudates, and whether this process mediates invasion in the field study.

## 5 Conclusions

This study provides evidence that PE microplastics can enhance the growth of both alien and native plants and influence the allelopathic potential of native plants exclusively, with direct implications for interspecific competition and ecosystem dynamics. The alteration of metabolic profiles in native plants following PE exposure may provide a novel approach to the management of invasive species, enhancing native plant allelopathy. Furthermore, more research is needed to fully understand the long-term ecological consequences of microplastics exposure and to develop strategies for mitigating potentially negative impacts on native plant communities.

## Data Availability

The raw data supporting the conclusions of this article will be made available by the authors, without undue reservation.

## Supplementary material

Supplementary Figure S1 Hierarchical clustering heatmap of the metabolomic differences in *Achyranthes bidentata* in the presence or absence of PE powder, expressing the relative abundance of metabolites with different colors.

Supplementary Table S1 Information on the five invasive and five native herbaceous species seeds used to determine the potential allelopathic effects of aqueous plant leachates on germination. Information on the number of Chinese regions (*n* = 34) in which a species occurs is adopted from Yan et al. (2019) for alien species, and is adopted from *Flora of China* (http://www.efloras.org/flora_page.aspx?flora_id=2) for native species. Information on the alien status and its invasiveness grade in China is achieved from Yan et al. (2014): Alien-1 = malignant invasive plant (i.e. alien species having significant and serious impact on the economy or environment), Alien-4 = alien plant that is common but does not have obvious impacts). Information on the year of first record of alien species is adopted from the Chinese Virtual Herbarium (https://www.cvh.ac.cn/). Information on the region of origin is from POWO (2019). Information on life cycle is obtained from the Flora of China.

Supplementary Table S2 The top 20 DAMs in *Achyranthes* after treatment with PE microplastics.

Supplementary Table S3 Results of a linear mixed-effect model used to test the competitive responses of plants when grown in the presence or absence of soil microplastics. The competitive responses were quantified using relative interaction intensity index (RII) values, which were calculated according to the above-ground biomass of the plants at the time point of harvest.
