# Supplementary figures and images for: Effect of microplastics on the allelopathic effects of native and invasive plants on co-occurring invaders
Source: Front Plant Sci. 2024 Oct 28;15:1425815. doi: 10.3389/fpls.2024.1425815 (PMC11551022; doi:10.3389/fpls.2024.1425815)

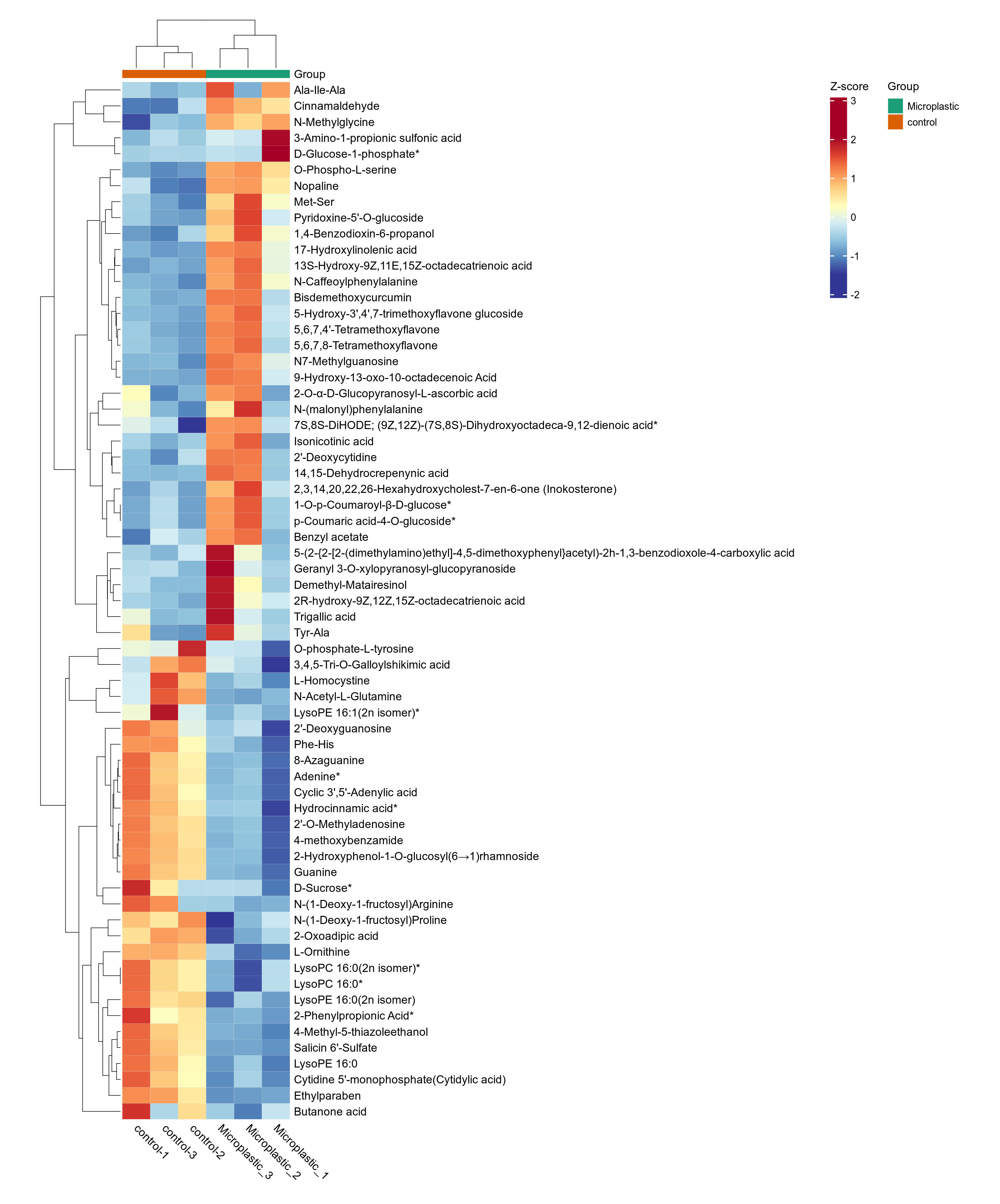

Supplement: Supplementary Figure S1 — Hierarchical clustering heatmap of the metabolomic differences in Achyranthes bidentata in the presence or absence of PE powder, expressing the relative abundance of metabolites with different colors. [file Image1.jpg]
