# Supplementary material for: Effect of microplastics on the allelopathic effects of native and invasive plants on co-occurring invaders
Source: Front Plant Sci. 2024 Oct 28;15:1425815. doi: 10.3389/fpls.2024.1425815 (PMC11551022; doi:10.3389/fpls.2024.1425815)
Supplement: Supplementary Table S1 — Information on the five invasive and five native herbaceous species seeds used to determine the potential allelopathic effects of aqueous plant leachates on germination. Information on the number of Chinese regions (n = 34) in which a species occurs is adopted from Yan et al. (2019) for alien species, and is adopted from Flora of China (http://www.efloras.org/flora_page.aspx?flora_id=2) for native species. Information on the alien status and its invasiveness grade in China is achieved from Yan et al. (2014): Alien-1 = malignant invasive plant (i.e. alien species having significant and serious impact on the economy or environment), Alien-4 = alien plant that is common but does not have obvious impacts). Information on the year of first record of alien species is adopted from the Chinese Virtual Herbarium (https://www.cvh.ac.cn/). Information on the region of origin is from POWO (2019). Information on life cycle is obtained from the Flora of China. [file Table1.docx]

Table S1. Information on the five invasive and five native herbaceous species seeds used to determine the potential allelopathic effects of aqueous plant leachates on germination.

| **Species** | **Family** | **Number of**  **Chinese**  **regions** | **Status in eastern China (year of first record)** | **Region of origin** | **Life cycle** | **Seed source** |
| --- | --- | --- | --- | --- | --- | --- |
| *Sesbania cannabina* (Retz.) Poir. | Fabaceae | 26 | Alien-1 (1910) | Indian Subcontinent to Indo-China, Australia | Annual | Junjie Nursery Stock Company (Jiangsu,Suqian) |
| *Amaranthus spinosus* L. | Amaranthaceae | 25 | Alien-1 (1905) | Mexico to Tropical America | Annual | Jiangsu Qixiu Seed Industry Co., Ltd (Jiangsu,Suqian) |
| *Lolium perenne*L. | Poaceae | 32 | Alien-4 (1905) | Europe | Perennial | Jiangsu Daxin Ecological Technology Co., Ltd (Jiangsu,Suqian) |
| *Capsella bursa-pastoris* (L.) Medik. | Brassicaceae | 34 | Alien-4 (1906) | Europe, and parts of Asia and Africa | Annual | Field in Taizhou |
| *Sphagneticola trilobata* (L.) Pruski | Asteraceae | 5 | Alien-1 (1997) | Mexico to S. Tropical America and Trinidad | Perennial | Junjie Nursery Stock Company (Jiangsu, Suqian ) |
| *Achyranthes bidentata* Blume | Amaranthaceae | 14 | Native | Eastern and Tropical Asia | Perennial | Thousand Green Seed Company (Jjiangsu,Suqian) |
| *Crepidiastrum sonchifolium* (Maxim.) Pak & Kawano | Asteraceae | 19 | Native | Eastern Asia | Annual/biennial | Thousand Green Seed Company (Jjiangsu,Suqian) |
| *Orychophragmus violaceus* (L.) O.E.Schul | Brassicaceae | 15 | Native | N.&E. China to N. Korea | Annual/biennial | Jiangsu huazhiyin seed industry (Jiangsu,Suqian) |
| *Arthraxon hispidus* (Thunb.) Makino | Poaceae | 20 | Native | Tropical Africa, W. Indian Ocean,Asia to E. Australia | Annual | Field in Taizhou |
| *Aeschynomene indica* L. | Fabaceae | 21 | Native | Tropical & Subtropical old world | Annual | Thousand Green Seed Company (Jjiangsu,Suqian) |
|  |  |  |  |  |  |  |

Information on the number of Chinese regions (*n* = 34) in which a species occurs is adopted from Yan et al. (2019) for alien species, and is adopted from *Flora of China* (<http://www.efloras.org/flora_page.aspx?flora_id=2>) for native species. Information on the alien status and its invasiveness grade in China is achieved from Yan et al. (2014): Alien-1 = malignant invasive plant (i.e. alien species having significant and serious impact on the economy or environment), Alien-4 = alien plant that is common but does not have obvious impacts). Information on the year of first record of alien species is adopted from the Chinese Virtual Herbarium (<https://www.cvh.ac.cn/>). Information on the region of origin is from POWO (2019). Information on life cycle is obtained from the Flora of China.

**References**

POWO. (2019). Plants of the world online. Facilitated by the Royal Botanic Gardens, Kew. Published on the Internet; http://www.plantsoftheworldonline.org/. Accessed 11 Jan 2021

Yan, X., Liu, Q., Shou, H. et al. (2014). The categorization and analysis on the geographic distribution patterns of Chinese alien invasive plants. Biodiv. Sci. 22, 667–676.

Yan, X., Wang, Z., Ma, J. (2019). The checklist of the naturalized plants in China. Shanghai scientific and technical publishers, Shanghai
