# Supplementary material for: Effect of microplastics on the allelopathic effects of native and invasive plants on co-occurring invaders
Source: Front Plant Sci. 2024 Oct 28;15:1425815. doi: 10.3389/fpls.2024.1425815 (PMC11551022; doi:10.3389/fpls.2024.1425815)
Supplement: Supplementary Table S2 — The top 20 DAMs in Achyranthes after treatment with PE microplastics. [file Table2.docx]

**Table S2.** The top 20 DAMs in *Achyranthes* after treatment with PE microplastics

| Compounds | Class I | Microplastic | control | Log_2_FC | Type |
| --- | --- | --- | --- | --- | --- |
| Demethyl-Matairesinol | Lignans and Coumarins | 104285 | 24236 | 2.11 | up |
| 5-Hydroxy-3',4',7-trimethoxyflavone glucoside | Flavonoids | 1643806 | 421323 | 1.96 | up |
| 9-Hydroxy-13-oxo-10-octadecenoic Acid | Lipids | 772912 | 216260 | 1.84 | up |
| 13S-Hydroxy-9Z,11E,15Z-octadecatrienoic acid | Lipids | 22946 | 7614 | 1.59 | up |
| D-Glucose-1-phosphate* | Others | 384425 | 128559 | 1.58 | up |
| 14,15-Dehydrocrepenynic acid | Lipids | 186015 | 64572 | 1.53 | up |
| N-Methylglycine | Amino acids and derivatives | 2160698 | 755613 | 1.52 | up |
| Bisdemethoxycurcumin | Phenolic acids | 137652 | 48954 | 1.49 | up |
| N-Caffeoylphenylalanine | Amino acids and derivatives | 160718 | 57648 | 1.48 | up |
| 2'-Deoxycytidine | Nucleotides and derivatives | 81863 | 29715 | 1.46 | up |
| N7-Methylguanosine | Nucleotides and derivatives | 343376 | 125276 | 1.45 | up |
| 17-Hydroxylinolenic acid | Lipids | 123408 | 45104 | 1.45 | up |
| 2-Oxoadipic acid | Organic acids | 83427 | 230808 | -1.47 | down |
| LysoPE 16:0 | Lipids | 8102 | 23216 | -1.52 | down |
| Guanine | Nucleotides and derivatives | 1171780 | 3360195 | -1.52 | down |
| 4-Methyl-5-thiazoleethanol | Others | 520898 | 1636147 | -1.65 | down |
| N-Acetyl-L-Glutamine | Amino acids and derivatives | 695123 | 2566905 | -1.88 | down |
| Ethylparaben | Phenolic acids | 18517 | 73334 | -1.99 | down |
| L-Ornithine | Amino acids and derivatives | 14102 | 60457 | -2.10 | down |
| Salicin 6'-Sulfate | Phenolic acids | 64842 | 690734 | -3.41 | down |
