# Supplementary material for: Effect of microplastics on the allelopathic effects of native and invasive plants on co-occurring invaders
Source: Front Plant Sci. 2024 Oct 28;15:1425815. doi: 10.3389/fpls.2024.1425815 (PMC11551022; doi:10.3389/fpls.2024.1425815)
Supplement: Supplementary Table S3 — Results of a linear mixed-effect model used to test the competitive responses of plants when grown in the presence or absence of soil microplastics. The competitive responses were quantified using relative interaction intensity index (RII) values, which were calculated according to the above-ground biomass of the plants at the time point of harvest. [file Table3.docx]

Table S3. Results of a linear mixed-effect model used to test the competitive responses of plants when grown in the presence or absence of soil microplastics. The competitive responses were quantified using relative interaction intensity index (RII) values, which were calculated according to the above-ground biomass of the plants at the time point of harvest.

| Factor | RII based on aboveground biomass of *Achyranthes* | | RII based on aboveground biomass of *Amaranthus* | |
| --- | --- | --- | --- | --- |
|  | F | *P* | F | *P* |
| Microplastic | 0.679 | 0.419 | 0.293 | 0.594 |
